# Fixed Drug Eruption of the Penis Secondary to Sulfamethoxazole-Trimethoprim

**DOI:** 10.1100/tsw.2006.361

**Published:** 2006-01-29

**Authors:** Nathan Lawrentschuk, David Pan, Andrew Troy

**Affiliations:** University of Melbourne, Department of Surgery and Urology and Department of Medicine, Austin Hospital, Studley Road, Heidelberg, Victoria, 3084, Australia

**Keywords:** drug eruptions, penis, skin, dermatology, urology

## Abstract

Penile lesions are encountered in a variety of fields from family medicine practice through urology, to sexual health specialists. It is important that practitioners consider and recognize fixed drug eruptions of the penis while being able to initiate appropriate treatment in order to avoid misdiagnosis and avoidable stress. In summary, withdrawal of the offending medication and initiation of corticosteroid therapy remain the cornerstones of treatment of fixed drug eruptions of the penis.

## CASE HISTORY

A 58-year-old male presented with sudden onset of a painless, swollen penis with blistering lesions. There were no associated systemic or urinary tract symptoms and there was no relevant past history, particularly of recent trauma or pruritus in the genital region. He had a stable sexual partner and no history of a sexually transmissible disease. However, he had been on an oral sulfur-containing antibiotic (sulfamethoxazole 800 mg-trimethoprim 160 mg, taken twice daily) for 48 h after being diagnosed with cellulitis of the hand.

On examination, his penis was edematous (Fig. 1) with lesions on the glans and distal shaft (Fig. 2). A broken lesion was swabbed with microscopy yielding no bacteria or polymorphs. Urine cultures were clear. After thorough history and examination, a fixed drug eruption appeared the most likely diagnosis. The antibiotic was ceased and topical corticosteroid (mometasone furoate; 0.1% in 15 g) was applied twice daily. He was reviewed the next day with the lesions and edema subsiding. All cultures were negative for bacteria. Three weeks later the lesions had completely resolved (Fig. 3).

## DISCUSSION

Fixed drug eruptions of the penis are rare, but have been reported[1,2,3,4]. Patients with such eruptions usually present with a sudden onset of single or multiple well-defined circular plaques on the glans penis and distal shaft of the penis. The eruption may be bullous and its surface can become necrotic and painful[5].

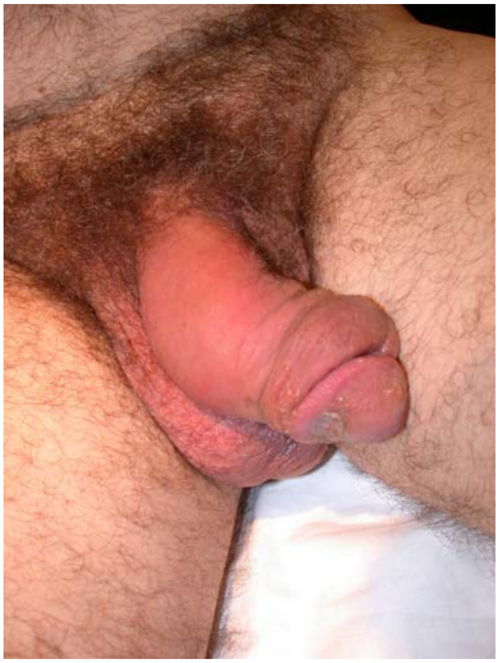

**FIGURE 1.** The swollen edematous penis with lesions on the distal shaft and glans penis.

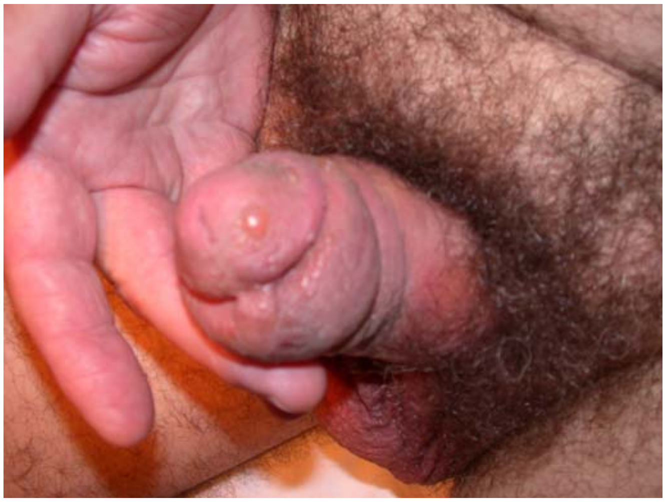

**FIGURE 2**. A closer view of the blistering lesions on the glans and distal shaft.

Sulfur is a drug known to cause a variety of allergies, including dermatoses. In a study of 113 patients with fixed drug eruptions of the skin, sulfamethoxazole-trimethoprim (co-trimoxazole) caused over a third[6]. Penile drug eruptions secondary to co-trimoxazole have been reported[7]. Ironically, sulfur-containing drugs potentially treat many dermatoses[8]. Other drugs more commonly associated with fixed drug eruptions of the penis include tetracyclines, acetaminophen (paracetamol), and laxatives containing phenolphthalein. In total, more than 500 medications have been implicated in fixed drug eruptions of the skin[5].

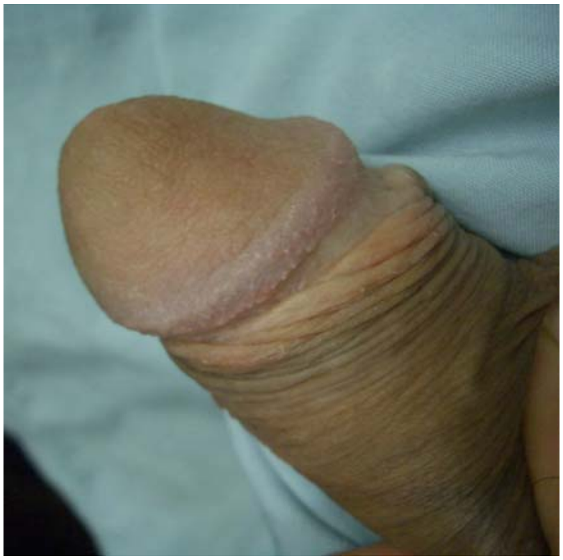

**FIGURE 3**. Resolution of the fixed drug eruption after treatment with corticosteroids.

As fixed drug eruptions are a diagnosis of exclusion, differential diagnoses that need to be excluded include malignancy and precancerous lesions (e.g., erythroplasia of Queyrat), infections (e.g., genital herpes, syphilis), balanitis, dermatoses (e.g., lichen planus, solitary plasmocytoma), trauma, and genital pruritus[9,10]. Biopsy or culture should be considered where a persistent eruption exists to exclude malignancy or confirm another diagnosis[5].

The key to managing the fixed drug eruptions of the penis is a thorough history with particular note of medications used intermittently, such as antibiotics for chronic prostatitis and analgesics (e.g., acetaminophen), while also excluding other conditions[5]. Once diagnosed, offending medications must be ceased and topical or oral corticosteroids commenced[6]. Most cases are then self-limiting and resolve. Patients should take note of drugs causing any allergies and these should be clearly documented in patient records to prevent future events. Multiple recurrences of fixed drug eruption may result in postinflammatory hyperpigmentation[5].

In summary, fixed drug eruptions of the penis, although rarely reported, are not uncommon and clinicians need to consider it as a diagnosis. Other diagnoses are excluded by taking a thorough history and occasionally biopsy or culture while treatment of the eruption involves withdrawing the offending medication and use of steroids.

## BIOSKETCHES

**Nathan Lawrentschuk, MB BS,** Urology Research Fellow, University of Melbourne, Department of Surgery and Department of Urology, Austin Hospital, Studley Road, Heidelberg, Victoria, 3084, Australia.

**David Pan, MB BS,** Urology Resident, University of Melbourne, Department of Surgery and Department of Urology, Austin Hospital, Studley Road, Heidelberg, Victoria, 3084, Australia.

**Andrew Troy, FRACS,** Urologist, University of Melbourne, Department of Surgery and Department of Urology , Austin Hospital, Studley Road, Heidelberg, Victoria, 3084, Australia.
